# General practice staff and patient experiences of a multicomponent intervention for people at high risk of poor health outcomes: a qualitative study

**DOI:** 10.1186/s12875-023-02256-6

**Published:** 2024-01-09

**Authors:** Sara Javanparast, Leigh Roeger, Richard L. Reed

**Affiliations:** College of Medicine and Public Health, Flinders University, Adelaide, Australia

**Keywords:** Australia, Health services, Continuity of care, GP appointment length, GP follow-up, Qualitative study, Chronic disease

## Abstract

**Background:**

This study reports the experiences of general practice staff and patients at high risk of poor health outcomes who took part in a clustered randomised controlled trial of a multicomponent general practice intervention. The intervention comprised patient enrolment to a preferred General Practitioner (GP) to promote continuity of care, access to longer GP appointments, and timely general practice follow-up after hospital care episodes. The aims of the study were to better understand participant’s (practice staff and patients) perspectives of the intervention, their views on whether the intervention had improved general practice services, reduced hospital admissions and finally whether they believed the intervention would be sustainable after the trial had completed.

**Methods:**

A qualitative study design with semi-structured interviews was employed. The practice staff sample was drawn from both the control and intervention groups. The patient sample was drawn from those who had expressed an interest in taking part in an interview during the trial and who had also experienced a recent hospital care episode.

**Results:**

Interviews were conducted with 41 practice staff and 45 patients. Practice staff and patients expressed support for the value of appointments with a regular GP and having sufficient time in appointments for the provision of comprehensive care. There were mixed views with respect to the extent to which the intervention had improved services. The positive changes reported were related to services being provided in a more proactive, thorough, and systematic manner with a greater emphasis on team based care involving the Practice Nurse. Patients nominated after hours care and financial considerations as the key reasons for seeking hospital care. Practice staff noted that the intervention would be difficult to sustain financially in the absence of additional funding.

**Conclusions:**

The multicomponent intervention was supported by practice staff and patients and some patients perceived that it had led to improvements in care.

## Introduction

An efficient and adequately resourced primary health care sector is recognised as critical for improved population health outcomes and for health funding to be sustainable. Australia faces an aging population, rising rates of chronic and complex disease and a growing demand for hospital and other expensive healthcare services. These challenges are not unique to Australia and have encouraged policy makers both locally [1] and internationally to consider wide ranging reforms to the organisation and funding models of their primary care sector. These primary care reforms have included improved access to and continuity of care (typically through patient registration), expanding multidisciplinary care and better coordination and integration between primary and hospital care [2].

In Australia general practice is funded by the federal government on a fee-for-service basis with most services eligible for rebates from the universal health insurance scheme known as Medicare. In contrast to other countries [3] where patients register to a particular GP or to a specific practice Australian patients are free to consult with multiple GPs, including those at different general practices [4]. Despite the lack of a formal registration system most Australians with (85%) or without (70%) a long term health condition report having a preferred GP [5] although multiple practice attendance, particularly for younger people, is not uncommon [6].

A strength of the Australian model is that promotes patient choice and encourages provider competition but the lack of a formalised relationship between GPs and their patients does not encourage continuity of health care which is widely recognised as a core principle of primary care [7]. Systematic reviews suggest that there is an association between continuity of care and patient satisfaction and health service utilisation [8, 9] but this evidence has mainly been generated from cross-sectional studies with very few interventional trials. Observational studies from countries that have introduced primary care registration systems to promote continuity of care are suggestive of better patient outcomes and reduced costs but overall the evidence base is considered weak [3].

The Australian fee-for-service funding model incentivises GPs to provide a higher volume of services as opposed to providing higher value care [10, 11]. In addition, the Medicare rebate structure discourages longer consultations by setting decreasing (on a per minute basis) patient rebates as appointment length increases encouraging GPs to focus on speed over need [12]. By international standards Australia’s average length of GP consultation of just under 15 min [13] would not be considered short [14]. The interpretation of this however is complicated by the fact that Australia’s fee for service model has acted as a barrier for the development of general practice multidisciplinary teams [15]. As a result, compared to GPs in other countries, Australian GPs have less opportunity to delegate administrative tasks and basic clinical work [12].

While general practice is funded and managed by the federal government, hospital services are administrated separately by the eight State and Territory governments. This provides little incentive for intersectoral collaboration or communication to deliver the best possible patient care. The transition between hospital to home and GP care is associated with high risk for adverse events and avoidable hospital readmissions, particularly for older people with complex needs [16–18]. The extent to patients receive timely GP follow-up after hospital discharge depends on a set of complex factors relating to patient’s perceptions of the value of GP follow-up, GPs receiving notification of the hospitalisation and hospitals communicating the need for GP follow-up to both the patient and the GP [19]. From the limited Australian research available it appears that in the order of around one-third of patients discharged from hospital do not see a GP within 14 days [19–21].

In 2018 the Royal Australian College of General Practitioners, in collaboration with the federal government, funded a clustered randomised trial (titled the Flinders Quality Enhanced general practice Services Trial: Flinders QUEST) to test a multicomponent general practice intervention aimed at improving health outcomes and health service use for patients at high risk of poor outcomes. The multicomponent intervention was designed to improve continuity of care (defined as general practice appointments with patient’s regular preferred GP), access to long appointments and timely (with 7 days) general practice follow-up if patients experienced a hospital care episode.

Flinders QUEST was conducted in 20 general practices located in the metropolitan area of Adelaide, South Australia. Ninety-two participating GPs were provided a list of their active patients (three or more visits in the previous two years) drawn from three cohorts (1) children and young people aged 0 to 17 years; (2) adults aged 18–64 with two or more chronic illnesses; (3) older people aged 65 years and above. GPs were asked to identify 18 patients who they believed were at risk of poor health outcomes and who may potentially benefit from the intervention. GPs were asked to use their clinical judgement to prioritise patients who were not too low a risk of poor health outcomes nor those who were so seriously ill that it was too late for the intervention to work.

The implementation of the intervention and quantitative results, at 12-month outcome assessment, have been reported elsewhere [22]. In brief the intervention was implemented to a reasonable standard and there were statistically significant improvements to continuity of care and the number of longer length of appointments. There was a greater likelihood of follow-up after emergency department or hospital care episodes but this was not statistically significant. The intervention was not found to improve self-rated health (the primary outcome of the trial), nor were there any statistically significant intervention effects for health service utilisation.

The economic evaluation found that the intervention was more effective in terms of Quality Adjusted Life Years (QALY) but considering the payment (A$1,000 per patient) intervention practices received for providing the intervention the intervention was not cost-effective. In a pre-specified exploratory sub-group analysis of older people (69% of the total sample), the intervention was found to be cost-effective primarily due to a reduction in hospital usage.

There is increasing recognition of the potential benefits of qualitative research within randomised clinical trials [23, 24] and Flinders QUEST included a qualitative component to complement the quantitative evaluations. Consistent with the most frequent use of qualitative research in clinical trials, particularly for complex interventions [25], the present qualitative study was focussed on the multicomponent intervention. Specifically, we wished to better understand participants perspectives on each of the components of the intervention, gather their opinions on whether the intervention had improved general practice services and may have resulted in hospital avoidance and whether practice staff believed the intervention would be sustainable after the trial had completed. It was intended that the findings from the qualitative study would complement the and enrich the understanding of the main quantitative results from the trial.

## Methods

This article was written in accordance with the standards for reporting qualitative research (SRQR).

### Context and setting

This study was conducted with general practice staff (Practice Managers [PMs], GPs, Practice Nurses [PNs) and their patients in Adelaide, South Australia.

### Study design

We conducted a qualitative study using semi-structured interviews. Separate interview guides were developed by the research team for practice staff and intervention group patients. Practice staff interviews explored perspectives of each of the components of the intervention (including factors that facilitated or constrained its implementation in practices), the mechanisms through which the intervention may have improved patient health outcomes and health service usage and finally the sustainability of the intervention after the trial had completed.

A draft interview guide was created by the first author (SJ) and then developed iteratively with the input from the Flinders QUEST chief investigator (RR) and trial manager (LR). The guide was designed to elicit participants perspectives (GP staff and patients) about the components of the intervention and whether the intervention had (in their opinion) improved general practice services (relating to continuity of care, appointment length and general practice follow-up after a hospitalisation) during the trial period. A key secondary outcome of the trial was whether the multicomponent intervention resulted in reduced hospital use. In the trial this was assessed quantitively from hospital administrative records and in the qualitative interviews we wished to better understand the mechanism through which any reduction might have occurred. For this reason we specifically targeted patients who had experienced one or more hospital care episodes during the intervention period. Given the small number of children and young people in the trial they were not included in the qualitative study.

### Participants

For general practice staff, we aimed to conduct one interview with the PM in each control group practice and three interviews (PM, GP, PN) in each intervention group practice. We included control group PMs because we were interested in their experience of the research process (e.g. patient recruitment, data provision, working with the research team) and also whether control group practices had engaged in any other quality improvement activities during the intervention period. For intervention group practices, we invited the PM to nominate a GP and a PN who had played an active role in the trial to take part in the interviews. A participant information sheet and consent form were forwarded to practice staff and the interview sessions arranged.

Of the 10 control group practices all PMs (one PM was the manager at two practices) agreed to be interviewed. Of the 10 intervention group practices all the PMs and their nominated GPs and PNs agreed to be interviewed. In one intervention group practice two PNs who had been involved in the implementation of the study expressed a desire to be interviewed jointly. In another intervention group practice an Administrative Officer who had played a key role in the implementation of the study was interviewed along with the PM at the PM’s request.

Patient recruitment to the qualitative study was conducted in three stages. Overall, 1044 patients drawn from three cohorts took part in Flinders QUEST: children and young people under the age of 18 years (*n* = 58); adults aged between 18 and 64 years with two or more chronic diseases (*n* = 315) and older people aged 65 years and above (*n* = 671). In the first stage, intervention group patients (in the adults and older adults cohorts) who had indicated a willingness via a response to a question in a six month follow-up questionnaire to take part in an interview about their experiences in the trial were identified. From 468 potentially eligible patients 391 (84.6%) had responded positively to this question. This was further refined to 188 (47.5%) patients who had reported one or more hospital care episodes (emergency department presentation or hospital admission) during the preceding 12 months. A purposeful sample of 55 patients was selected to ensure representation by practice, cohort (adults with two or more chronic diseases and older people aged > 64 years) and gender. An initial phone call was made to potential interviewees to provide background information about the qualitative study and for those expressing an interest in receiving further details a participant information sheet and consent form was posted to them. Of the 55 patients approached, 45 agreed to be interviewed with written consent completed at the time of the interview. Patients received a A$20 gift voucher for participating in the interview.

### Data collection

The practice staff and patient interviews were conducted by the first author, an experienced qualitative researcher, between November 2019 and March 2020, which was after the 12-month intervention period of the trial had completed. Practice staff interviews were conducted in general practices and took between 30 and 45 min. The patient interviews mostly occurred in patient’s homes and took between 30 and 60 min. The study was not affected by COVID-19; the first recorded case in South Australia was reported on 1 February 2020, by which time most of the interviews had been completed.

### Data analysis

The interviews were audio-recorded for transcription and further analysis. Interview files were transcribed by a professional transcribing service and imported to the qualitative analysis software (NVivo 12). A coding framework was developed using the key themes that emerged from the interviews and discussed in the research team.

## Results

Forty-one face-to-face interviews were conducted with practice staff (19 PMs, 1 Administrative staff, 11 PNs and 10 GPs). Forty-five interviews (3 by phone and 42 face-to face) were conducted with patients (25 female and 20 male). Most patients interviewed were drawn from the trial’s older adults cohort (69.0%) and the overall mean age was 71 years (SD = 11.3). Most patients were married (73.3%), retired (60.0%) and had a yearly income of less than A$60,000 (63.6%). Patients reported a mean of 3.9 (SD = 1.6) chronic diseases with the most frequent types being cardiovascular (57.8%) and musculoskeletal (68.9%) disorders. At baseline (the start of the intervention) patients reported a mean of 2.5 (SD = 2.6) emergency department presentations and 1.4 (SD = 1.9) hospital admissions during the preceding 12 month period.

### Practice staff and patient perspectives of the multicomponent intervention

#### Continuity of care

Practice staff valued GP continuity of care for service quality, trust and building relationships:

*“Continuity of care is that you don’t have to cover off that big chunk of their life every time. You just know what’s going on, just by them walking in the room before they even said anything…You get that connection with people. They will always tell you more with that connection. You’ve got more time because you don’t have to cover all that other history that you already know. Yeah, you can kind of nuance things a bit more, because you can pick the little things out that aren’t quite right with what’s going on.”* (Intervention practice, GP).

While continuity of care with a regular GP was generally supported, some practice staff drew a distinction between patients being ‘loyal to the practice’ versus being ‘loyal to a regular GP’. Strategies such as communication between GPs within a practice, systems in place enabling GPs to share patient’s notes and information and having a second regular GP were viewed by some as more likely to lead to a sustainable general practice model:

*“I should be selling the practice, the quality practice where all the doctors write good notes and knowledgeable caring and are on the same page. That’s what I’d like to do is sell the practice rather than an individual GP because if I go away for three weeks or something then what do they do? We all have holidays.”* (Intervention practice, GP).

There were also perceived clinical benefits from having a variety of different GPs involved in patient care:

*“I don’t want any of my patients to be absolutely dependent on me. I actually think it’s healthy for them to see another doctor because I might miss something that another doctor picks up. I might have some level of expertise in one area and another doctor might have a level of expertise in another area, so I actually think that concept of a preferred GP needs modification.”* (Intervention practice, GP).

A PM also commented:

*“You sometimes get a patient so dependent on one doctor that if that doctor’s away, they won’t see anyone else. To me, that’s putting their health at risk. It’s hard sometimes to convince people that, ‘Yes, even though he’s away, please come and see’ – and sometimes another doctor can shine a different light on the problem.”* (Control practice, PM).

Continuity of care was valued by patients for largely the same reasons as practice staff. Patients believed it enabled them to build trusted relationships with their GP and helped their GP to better understand their family and social circumstances:

*“Well, familiarity I guess and just I suppose the information, the things that you discuss with him then, I mean he’s got a fuller picture of a person as a patient, rather than just a random doctor here and there. They don’t have the whole picture.”* (Female, 75 yrs. Old).

Continuity of care was particularly valued by patients with complex health problems, mental illnesses or for other ‘personal’ issues:

*“I said to her [doctor] I didn’t feel like having to explain everything to everybody; especially when you’re losing blood from the bowel region, it’s embarrassing.”* (Male, 66 yrs. Old).

Several patients also raised the idea of a second regular GP or a practice-based GP service. For some, particularly those with long-term connections with their general practice, being able to see any GP within the practice was a way to fill in the gaps in care continuity with their preferred GP. Patients also reported the benefits of visits with other GPs, for example, consultations with a female GP for gender specific health issues or screenings or with GPs who are specialised in specific health conditions such as skin cancer. Patient perceived barriers to continuity of care included GPs who worked part-time, particularly, younger aged female doctors and GPs absences due to sickness.

#### Longer appointments

Longer appointments were viewed by practice staff as being particularly advantageous for patients with chronic and complex health conditions because they enabled more comprehensive care to be provided:

*“Rather than just looking at what the presenting complaint may be, it’s doing a full thorough check on someone. It’s being able to take that time to properly talk to them, to properly give education, to provide information, make sure that the understanding is there and answer any questions and that sort of thing, which is really important.”* (Intervention practice, PN).

On the other hand, some practice staff noted that patients with chronic and complex health conditions were more likely to have frequent appointments and this might be more beneficial compared with less frequent longer appointments:

*“If they’re seen once a month, a 15-minute appointment once a month that would be more valuable to them than a half-hour appointment every three months…and if they’re checking in with the nurse every three months, monthly 15-minute appointment with the doctor, they’re going to get everything that they need.”* (Intervention practice, PM).

Some GPs noted that the current Medicare rebate structure discourages longer appointments, and this requires changes at broader policy and system levels:

*“The way the schedule fee is based, you’re actually rewarded for shorter appointments rather than longer appointments. I think ultimately that needs to change, because the population at large is getting older, the complexity of patients is getting more difficult, and patients are becoming more knowledgeable. They don’t want to come in and just be given a script and told to do this or do that, they want more of an explanation of what’s going on and the rest of it.”* (Intervention practice, GP).

Longer appointments were valued by patients because it was felt they provided an opportunity to discuss complex health problems thoroughly (especially in the case of mental health issues), and to review medications. Longer appointment times were also thought to allow time for more questions to be asked. One patient described standard length appointments as:

*“… I think this business about working on a 15-minute appointment, to my mind, doesn’t work well, right…I call it shop medicine, right, you go in and you buy something and you go out.”* (Male, 89 yrs. Old).

However, patients also reported that appointment length should be based on need and that a long appointment was not always required for example for prescription renewals, test results or for simple problems:

*“Sometimes it’s not needed. Sometimes I think 30 minutes was too much, but there was other times when it was good to have that extra time.”* (Female, 58 yrs. Old).

Patients also raised the importance of doctor-patient communication as being equally important to appointment length:

*“What is important is the communication between the doctor and patient. You may have a longer appointment but if the doctor doesn’t communicate well, the longer appointment does not work.”* (Male, 69 yrs. Old).

#### Timely follow-up after a hospital care episode

Patient follow-up after a hospital care episode was considered by practice staff to be valuable but challenging to achieve consistently due to poor communication between hospitals and general practices which led to delays in practices receiving patient discharge summaries:

*“I think the biggest thing is that the discharge information, once they’ve been in hospital and struggling to pin people down.”* (Intervention practice, PM).

*“It really disrupts the continuity because eventually you’ll say, they need to have follow up bloods done three days post discharge. Well, they’ve been home for a week now.”* (Intervention practice, PN).

*“Private hospitals don’t have their own resident medical staff. And it’s a medical handover issue, they don’t see that as their problem. As opposed to a public hospital where they actually have a resident medical staff so you can address the concern or something to that staff as an entity. And that’s the problem I think, that’s where it breaks down unfortunately.”* (Intervention practice, GP).

Similarly, patients reported examples of their GP not being aware of their hospital care episode:

*“He [GP] said “When did you get back?” and I said “Mate, I’ve just been in hospital”… I told him! So you know, that’s where the system falls down.”* (Male, 65 yrs. Old).

Another patient reported:

*“I think there is a problem with the hospital and their follow-ups. Whenever I came out from a stay in hospital, I’d do a follow-up appointment with the doctors and usually they’d have no idea that you’ve been in there. They haven’t received follow-up reports or anything like that… the run of the mill is that you’re told you should follow-up within a fortnight of leaving hospital. So we’d make that appointment and you’d see them [GP] and they’d say “Oh we haven’t received anything” so there is a lag time there.”* (Male, 69 yrs. Old).

### Practice staff and patient views on the impact of the intervention on general practice services

The extent to which the intervention had provided general practice care that was different from usual care was one of the key themes that emerged from the practice staff interviews. Noting that the patients in the trial had been identified as at high risk of poor outcomes, practice staff often felt that they had strategies in place prior to the trial to ensure high levels of continuity of care and access to longer appointments:

*“It was along the same path of what we’ve already done, been trying to do something to help the target group in this case… If we managed to get into intervention, then the work that we do with our patients is not dissimilar to what we do already, in terms of that patient care and trying to give that extra bit which the QUEST was all about.”* (Control practice, PM).

*“We are already doing the job, but now we had more incentive, we’ve got appointments and more nurses and staff were knowing that we need to take care of people, those people better and more efficiently… Yeah, special treatment, it made it a bit more systematic.”* (Intervention practice, GP).

There was a common perception that the intervention had largely been a part of usual care and therefore there were not significant changes because of the trial:

*“As a practice, that’s how we like to run things anyway. We’re very chronic-diseased focused, which you’d want to see the same GP if you can, not always possible, but most of the time we will try and make that happen anyway.” (Intervention practice, PM)*.

*“We’re already implementing a lot of things before you even start. So to some degree it was a bit the icing on the cake.” (Intervention practice, GP)*.

On the other hand, some intervention group practice staff reported that participating in the trial had increased their level of awareness about proactively addressing the needs of high risk patients:

*“I’ve become more proactive with patients’ problems. Not only with the patients registered in the trial, but even with other patients coming in with similar problems. I initiated the same sort of practices so that they can benefit also, like giving them more time, looking into more preventative care before the problem started.” (Intervention practice, GP)*.

The benefits of longer appointment times were appreciated by some GPs who noted that:

*“It gives you time to not rush and be able to let them open up and talk about what’s going on, and look up their bloods and bone densities and all those kinds of things that in 15 minute-appointments, I find it to be too short, sharp. Because 15 is really 10 to 12, by the time you get notes and things done, it doesn’t give a lot of time. So, personally, double appointments I think are more beneficial, and reduce stress levels as well.”* (Intervention practice, GP).

As noted, earlier patient follow-up after a hospital care episode was viewed by practice staff as challenging to implement and efforts to encourage patients to inform the practice of their hospital episode not always successful:

*“ … we try to tell the QUEST patients to ring us, to get a relative to ring us if they’ve been hospitalised, but they don’t because you don’t think of it, and they assume that the hospital is telling them. They rock up here and say, “I’ve been in hospital,” and you go, “Really?”* (Intervention practice, PM).

There were contrasting views amongst patients on the impact of the intervention on the general practice services they received. Some patients believed that there had been little or minimal changes noticeable during the intervention period. Patients with chronic and complex health conditions believed that due to their special circumstances the practice had always offered priority services including longer GP appointments and regular check-ups:

*“It’s always been really good, that surgery, which is why I’ve been there for 20 years.”* (Male, 62 yrs. Old).

*“So it’s pretty hard to say that QUEST made him that way. I think it’s just in general, he’s just a good old fashioned GP who wants to spend time with the patient.”* (Male, 57 yrs. Old).

*“Because I’m so complex and one thing can happen and I’ll just drop and be really sick, I’ve always been well first priority. Yeah so I always feel looked after and there’s support there if I need it.”* (Female, 53 yrs. Old).

On the other hand, there were those who felt that general practice services improved during the intervention period. These improvements included the improvements to the waiting times for appointments, an increased awareness with participant’s health and healthcare and better access to long GP appointments:

*“ … prior to it (the trial) we did have a couple of occasions where he was fully booked up for a week. I never got that once we started the QUEST program, yeah. Whenever you’d ring up and say “Okay next available is?” “Is it urgent?” “Yeah” so you’d get in if not that day, the following day. It’s encouraged them to lift their game.”* (Male, 73 yrs. Old).

*“Being part of this program, I got enhanced medical treatment… I am really seriously ill and I guess this program allowed my surgery practice to actually streamline me.”* (Male, 66 yrs. Old).

*“QUEST trial has made him [doctor] more aware that he has to be quite thorough, even though he is thorough, but I think it is in the back of his mind.”* (Female, 74 yrs. Old).

*“I’ve felt less pressured to get in and get out. I’ve felt like it’s okay to come in, and take a breath, and say, “Okay, this is what I’m doing, and this is how I’m feeling… it’s been nice to be able to sit there with her and go, “Look, I’m coming down. I can feel my depression deepening.” To be free enough to talk to her about that without thinking, “I’ve got to be out of there in five minutes” So it’s been better in that respect.”* (Female, 50 yrs. Old).

The role of the PN in the trial was raised by several intervention group practice staff and patients. In some practices the PN had played a very limited role confined to patient recruitment while in other practices the PN had a more active role in implementing the intervention and this appeared to facilitate a more team based approach to care:

*“We trialled a pod kind of thing, so we allocated each patient with their own doctor, their own nurse, and their own admin team, so we tried to do the pod environment…we have a pharmacist on the team as well.”* (Intervention practice, PN).

This team-based approach was viewed as being a strong enabler to the successful implementation of the intervention as well as improving patient satisfaction and engagement:

*“I guess the QUEST patients loved having their nurse. They absolutely loved it. They take ownership. Like, “That’s my nurse.” … It was actually nice and then they’ve got someone that they can call. Sometimes they feel like they don’t want to bother the doctor, or it might be a silly problem. Then they know that they can just call up, have a conversation with the nurse.”* (Intervention practice, GP).

This model of team based care was viewed by practice staff as a more ‘*sustainable practice model’* that reduces ‘*burn-out’* and ensures continuity of care: ‘*continuity isn’t necessarily with the one person [doctor]. It’s with a team.’ (Intervention practice, GP)*.

Patients also noted a stronger involvement of PNs during the trial which was viewed positively:

*“The only change that I saw in there was with the nurse. – for the good, not bad. The nurse, you know, it came up, “You have an appointment with the nurse.” I go there and she take the blood pressure and measure the asthma thing, all the stuff. Some time I had appointment just with the nurse, and then she gathered up the stuff and put it in a folder, and they put it into the computer for the doctor. That was a new thing, that was a good thing.”* (Female, 77 yrs. Old).

*“But I must admit I have, since the QUEST program I suppose, I’ve got even more time with also a nurse, check my blood pressure and my weight and anything like that. And then I go and see her [doctor].”* (Female, 68 yrs. Old).

### Practice staff and patient views on the impact of the intervention on hospital service use

Overall, from the practice staff and patient interviews it was difficult to establish a direct link between the intervention and hospital care episodes. Indirectly it appeared that for practice staff their participation in the trial and the fact that they (GPs) had identified trial patients as at high risk of poor health outcomes raised awareness for the potential to reduce the risk of avoidable hospitalisations:

*“I’ve thought of those patients differently. When they came I used to think, what could lead you into hospital and how can we avoid that.” (Intervention practice, GP)*.

From the patient perspective, however, the most frequently cited reason for hospital care as opposed to a GP appointment was to seek after-hours care:

*“We did call the ambulance a couple of times but that was because of the hour of the day and they [general practice] weren’t working, you know, it was early in the morning or something.”* (Male, 69 yrs. Old).

*“Well, it’s often in the evening, I mean I know I’ve got access to the after-hours service, but how do I get there? That sort of thing, for me that’s an issue, my disability…much easier to call an ambulance. “*(Female, 63 yrs. Old).

Other perceptions included receiving more comprehensive care in hospitals including access to specialists, radiology or other services that are not available in general practices:

*“The surgery couldn’t help me, the GP will see you straightaway but sometimes it’s like you try and book in for a scan or X-rays and things like that, you might have a two-week waiting list now and that sort of scared me a bit. I was thinking, you know, if I’ve got a blockage or something like that, or a twisted bowel. They [at hospital] do it straightaway.”* (Female, 71 yrs. Old).

A few patients highlighted financial issues as an incentive for attending hospitals instead of visiting a GP:

*“I mean, you go in there [general practice] and before they’d even look at you you’ve got to pay up – money up front. Even if you’re a private patient or if you’re – whatever you are you’ve still got to pay them cash up front. Then you sit and wait and they come along and have a look at you and send you off for an X-ray and so you go to an X-ray and that costs you about another three or four hundred.”* (Male, 64 yrs. Old).

### Sustainability of the intervention

Intervention group practice staff were generally positively disposed to continuing to provide the intervention after the trial had completed but it was acknowledged that providing the intervention without the additional funding from the trial would be difficult.

For example, improvements to continuity of care (appointments with the preferred GP) had been facilitated during the trial by intervention group practices reserving appointment slots for trial participants. But if the appointment time was not booked this could financially disadvantage the GP and practice:

*“… we just don’t have free appointments to be able to hold a few back. Yeah, I don’t think it would be a sustainable thing for us. We would love to be able to do it, for the most vulnerable patients and have those appointments free to be able to do it, but financially doctors aren’t going to hold appointments back, just in case they don’t get booked.”* (Intervention practice, PM).

Similarly for long appointments, intervention group practices were asked not to charge co-payments to trial patients to ensure that patients were not financially disadvantaged when receiving long appointments which are often associated with higher co-payment charges. Practice staff reported that bulkbilling for long appointments would be challenging to sustain and as a result the number of long appointments would likely reduce:

*“We will continue with a lot of those interventions, as normal. But the double appointments might be a different story. But we do have patients that aren’t specific QUEST patients who it is highlighted to preferred double appointments, and that is based on acuity and how chronic they are and problems going on. So that practice may reduce somewhat with those extra, longer appointments*.” (Intervention practice, PN).

Practices that had used the trial funding to support a greater emphasis on team based care also indicated that this would be difficult to sustain financially:

*The funding will prevent us from doing so. We employed more nurses, so we’ve got more nursing staff, but if we can’t fund that then where do we go? But the nurses take the pressure off the doctors and the doctors are under enormous pressure, so you want to maintain your doctors, stop them burning out because that’s not good for anyone, and also make sure you’ve got patients coming in. The doctor workforce is going to be tighter and tighter, so operating a good practise where you look after them and keeping them is key, from a workforce point of view.” (Intervention practice, PM)*.

## Discussion

This study has reported the experiences of general practice staff and their patients who took part in a clustered randomised controlled trial of a multicomponent general practice intervention. The elements of the multicomponent intervention were broadly supported by practice staff and patients who recognised benefits for patient care from appointments with a regular GP, having sufficient time in appointments to discuss complex health problems and receiving timely general practice follow-up after hospital care episodes. That support however was not unqualified with practice staff raising potential advantages for the involvement of different GPs in a patient’s care and poorer outcomes for patients who might inappropriately delay treatment until they could make an appointment with their preferred GP.

From the patient perspective, particularly those with complex health problems, continuity of care was highly valued but at that same time it was appreciated that in practice it was unlikely that appointments were always going to be able to be made with one’s preferred GP. Both practice staff and patients recognised that not all GP appointments needed to be longer length and that for people with chronic illnesses there might be benefit from more frequently occurring pre-planned standard length appointments.

Providing timely (within 7 days) follow-up after patients experienced a hospital care episode was reported by practice staff as challenging. General practice follow-up after hospital care episodes involves complex dependencies between the patient, the GP and the hospital [19]. Practices rely on discharge information being sent to them from hospitals or patients independently notifying them of their hospital care episode. But even when informed, intervention group practices were required to take a proactive approach initially to assess whether a follow-up appointment was clinically warranted and then to contact the patient to discuss whether they wished to attend for a face-to-face appointment. From the patient perspective there appeared to be element of surprise that there was not a seamless transfer of information between hospitals and general practices and that they themselves shared some of the responsibility for informing their general practices of any hospital care episodes.

That intervention group practice staff experienced challenges ensuring timely follow-up of patients after a hospital care episode is not surprising given prior research both in Australia [26, 27] and internationally [28–30] identifying the timeliness and quality of discharge letters as potential sources of conflict between GPs and hospital doctors. From the GP and patient perspectives very clear follow-up instructions are required to facilitate the provision of appropriate care to patients after their discharge from hospital [31]. The assumption of a seamless transfer of information between hospital and general practices is a risky one, particularly given that systematic reviews of the quality of discharge letters have found that key components including information about follow-up and management plans are often lacking [26, 29]. In light of this initiatives designed to empower patients to have a better understanding of their follow-up recommendations through a simplified set of discharge instructions are likely to be of potential value [32].

Many practice staff and patients reported that the intervention did not provide markedly different care than usual. Practice staff indicated that for the patients in the trial, who had been identified as at high risk of poor health outcomes, they had existing strategies in place to ensure high levels of continuity of care and patients had always had ready access to longer appointments. Similarly, patients often did not report major changes from usual care, but some reported a higher degree of thoroughness taken by their GP, more timely appointments, being routinely offered longer appointments and a greater involvement of the PN in their care. These findings are consistent with the quantitative results from the process indicators collected during the trial that showed only limited improvements to continuity of care, appointment length, and hospital follow-up.

The role of the PN in the trial was variable. In some intervention group practices the PN role was confined to assisting with patient recruitment and data collection. In other practices the PN took on a coordinating role in the implementation of the intervention. For two intervention group practices the trial appear to act as a catalyst for a more formalised team based approach and there was an increase in the amount of contact time between patients and PNs. A team based approach generally was thought by practice staff to a be a more sustainable model for general practice. This is consistent with the view that team based care is a critical component for high quality primary care [33]. From the patient perspective the adoption of a more team based approach was viewed very positively and indeed for some patients, the most noticeable (and positive) change for them during the trial was increased level of PN care that they received. This finding is broadly consistent with prior Australian research showing that patients who attended practices where PNs worked with broad scopes of practice were more likely to be more satisfied than those attending practices where PNs worked with narrow scopes of practice and low levels of autonomy [34].

While a focus on team based care was not one of the four elements of the multicomponent intervention, the significant payments made to intervention group practices supported practices to increase the amount of PN care provided to trial patients. The trial payments were A$1,000 per intervention group patient and with an average of around 50 patients per practice this equated to average practice payments in the order of A$50,000 (noting that the two largest intervention group practices had around 100 patients each). These payments were addition to the usual Medicare fee for service that practices received. For the duration of the trial, for some patients at the practice (i.e. those in the trial), the trial payments reduced the dominance of the fee for service payment structure which has been identified as a barrier to implementing team-oriented primary care [15].

In the economic evaluation, the intervention was found to be cost-effective in a pre-specified exploratory analysis of older patients primarily due to a reduction in hospital usage. The practice staff qualitative interviews suggested that the act of identifying a sub-set of patients as at high risk of poor health outcomes and being aware that hospital was an important outcome in the trial heightened awareness to the risk of potentially avoidable hospitalisations for trial patients. The exact mechanism through which this heightened awareness may have translated to reductions in hospitalisations however is unclear.

From the patient perspective the most frequently cited reasons for hospital care as opposed to a GP appointment were to seek after-hours care and to receive a more comprehensive and timely level of care could be provided in the hospital setting. Patients also nominated financial considerations observing that public hospital services are usually provided at low (or nil) direct cost to patients. Notably these key patient-nominated drivers of hospital use were not addressed by the intervention.

Practice staff expressed a general desire to continue to provide the intervention after the trial completed but they also indicated that changes they had made to operationalise the intervention in their practice, such as reserving appointments for trial patients to facilitate continuity of care, would be difficult to sustain financially when the significant trial payments ceased. The general feeling of many practice staff interviewed was that the financial challenges associated with implementing practice change were often underappreciated by people outside the general practice setting.

Importantly the concerns by practice staff about the financial challenges associated with practice change were expressed just prior to the COVID-19 pandemic which appeared in South Australia within weeks of the completion of most of the qualitative interviews. The impact of COVID-19 on general practices across Australia was substantial and included a decrease in the number of face to face to consultations, the rapid adoption of telehealth consultations, workforce shortages and financial pressures [35, 36]. Chronic disease management is likely to have suffered during the early phases of the pandemic due to general practice service disruptions and patients with chronic diseases avoiding healthcare appointments to minimise the risk of contracting COVID-19 [37].

It is unknown how COVID-19 impacted on the individual patients and practice staff that were interviewed for the present study and whether post the initial phases of COVID-19 they may have different perceptions about the value of the intervention. The Australian government’s *Strengthening Medicare* report [38] published in April 2023 recommended a broad primary care reform agenda including several elements to the Flinders QUEST multicomponent intervention. These included encouraging GP continuity of care through the introduction of voluntary patient registration, additional funding for longer consultations, supporting multidisciplinary team-based care and better integration and coordination between primary and secondary care. The findings of the present study suggest that these reforms would enjoy broad support from patients and qualified support from general practice staff on the proviso that any reforms were adequately funded.

### Limitations

The key limitations of this study relate to the generalisability of the findings. The practice staff who took part in Flinders QUEST were from practices who were part of an academic practice research network and who at the time of trial had the capacity to make changes to their systems of care. In addition, the GPs and PNs interviewed were nominated by the Practice Manager and their views may not be representative of clinical staff at their practice. Trial patients were drawn from a general practice patient population at risk of poor health outcomes, and this led a high proportion of older people who tended to have a long term relationship with their practice and GP in the trial. For the interviews this was further refined to those who had reported a recent hospital care episode. Younger patients, those without a regular GP, and in good health, might expected to have experienced the intervention very differently. Finally, the intervention itself was designed around perceived weaknesses in Australia’s primary health care system and the findings reported here may not be applicable in other countries.

## Conclusions

The multicomponent intervention was supported by practice staff and patients who appreciated the value of GP continuity of care, longer GP appointment times and GP follow-up after a hospital care episode. Some patients reported an improvement in their care during the trial, but many did not notice significant changes from the intervention. Practice staff generally viewed the intervention as providing usual care but in a more systematic and rigorous manner. Practice staff expressed a desire to continue the intervention after the trial had completed but noted that it would be difficult to sustain financially.

## Data Availability

The datasets generated and analysed during the current study are not publicly available due to the potential for identifying participants. The datasets are available from the corresponding author on reasonable request.
